# Preoperative Topical Hypothermia used in Prolonged Severe Lower Limb Ischemia to Avoid Ischemic Damage - The First Clinical Experience

**Published:** 2013-09

**Authors:** Claes Forsell, Jonas Åberg, Zoltán Szabó

**Affiliations:** 1 Department of Cardiothoracic and Vacular Surgery and Cardiothoracic Anesthesia, Linköping University Hospital, Sweden;; 2 Division of Cardiovascular Medicine, Department of Medical and Health Sciences, Linköping University, Linköping, Sweden

**Keywords:** skeletal muscle, topical hypothermia, limb ischemia, ischemic damage, protection

## Abstract

Severe lower limb ischemia TASC IIB/III with sensory and motor neurologic deficiencies leads to prolonged hospital care, amputation, and death in 20-70 % of cases. We present our first clinical experience of the use of preoperative topical hypothermia to improve muscular viability in these patients. Two hours after onset of symptoms, six 4-liter plastic bags were filled with snow and packed against the ischemic leg which was protected from frost injury by a layer of towels. After surgical revascularization four hours later muscular and neural functions in the leg were completely restored. A maximum serum myoglobin of 6500 ng/L (median 12000 ng/L in similar but untreated patients) postoperatively decreased to 1400 ng/L after 27 hours.

## INTRODUCTION

Acute arterial occlusion of the leg is common. The incidence of severe limb ischemia requiring revascularization within 6-8 hours is 2-4 cases /100 000/year (Swedish Vascular Register 2008-2012). Severe ischemic symptoms with sensory and motor neurologic deficiencies (TASC typ IIB/III) are consistent with ischemic muscle/nerve damage leading to fasciotomy, long hospital care, renal- and multi organ failure, amputation and a 30-day mortality of 20-70 %.

In heart surgery topical hypothermia is still used for myocardial protection (1-3). There are only sporadic publications on the experimental use of topical hypothermia to protect skeletal muscle, for example: musculo-cutaneous flaps (4, 5); isolated gracilis muscle (6); and post-ischemic hypothermia to diminish ischemic damage (7, 8). A small animal model has also been used to study the question (8). However, we could not find any publications regarding the clinical use of topical hypothermia to protect limb muscles against severe ischemia pending revascularization.

We present a case report on the successful use of preoperative topical hypothermia of the lower limb to ameliorate ischemic damage in prolonged severe lower limb ischemia.

## CASE REPORT

A seventy year-old man with a history of hypertension, rectal and colon carcinoma, ileostomy and four days of intestinal flu, presented with sudden onset of intense pain in the right lower leg. At the primary hospital the leg was cold, pale and sensory and motor functions were impaired. Femoral arterial pulses were normal. The anterior tibial and posterior fibular pulses were absent on the right side. After telephone contact with the vascular surgeon on-call, 5000 U of unfractioned Heparin were given intravenously, and the patient urgently transferred to our department for revascularization. Duplex sonography showed an occlusion of the right femoral artery. After approximately two hours of ischemia, the patient had total loss of sensory and motor function of the lower leg and foot and marbling of the skin. The calf muscles were soft.

Since the leg was severely ischemic, and some delay in surgery was expected in order to treat severe hyperkalemia, the idea to cool the leg to ameliorate ischemic damage came up.

**Figure 1:**
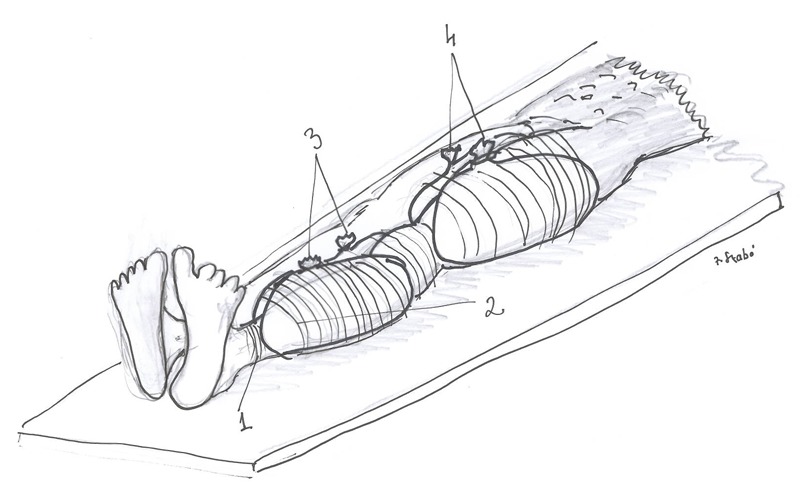
Schematic presentation of the “cool bags” round the left leg. 1 is the first layer of towels under the snow-bags to protect the limb against the freeze injury; 2 is the second layer of towels on the bags round the limb; 3-4 are the four snow bags used to cool the limb muscles.

*The procedure:* Six 4-liter plastic bags were each filled with snow and packed against the ischemic leg that was protected from frost injury by a layer of towels. A second layer of towels were packed over this as shown in Figure 1.

Preoperative assessment revealed dehydration with raised levels of hemoglobin (157 g/L), creatinine (547 mMol/L) and potassium (5.4 mMol/L). Diuresis prior to arrival at the vascular surgery department had not been measured. Severe hyperkalemia was treated with oral Resonium^®^ and an infusion of glucose and insulin (Insulin 5IE/h; 5% glucose +40 mMol Na 200 mL/h). The surgical procedure was performed under general anesthesia six hours after the onset of pain and 4 hours after application of the cooling bags. Circulating volume was optimized with cristalloids and albumin. Forced diuresis was achieved using mannitol and furosemide. Metabolic acidosis was buffered by hyperventilation and Tribonate^®^. A maximum S-Lactate of 2.9 mmol/L was found at the end of the operation. Diuresis during surgery was 1200 mL/2 h.

The operation was carried out via a lateral longitudinal incision. The thrombo-emboli were removed through a longitudinal arteriotomy of the profunda femoris artery. There was no backflow from the superficial femoral artery (SFA) and no embolic material was retrieved with the Fogarty catheter. The arterectomy was closed with a running 5.0 prolene suture without a patch, and flow was restored after a bolus dose of 200 mL of iv. Mannitol. Using a Doppler pen, a good flow signal was heard from the profunda artery but not from the SFA. Complete fasciotomies of all compartments were made through long medial and lateral incisions. The muscles were pale with some reaction to electocautery. No Doppler flow signals were heard from the anterior and posterior tibial arteries exposed in the incisions. Through small transverse arteriotomies, embolectomy was carried out in both of these arteries. After removal of the thrombotic material a brisk inflow and some backflow was obtained. After suture of the arteriotomies with interrupted 7.0 prolene there were good pulse and flow signals, muscles and skin of the foot were hyperemic. The fasciotomy incisions were left open with a loose running intracutaneus 2.0 prolene suture rather like an untied shoestring.

**Figure 2:**
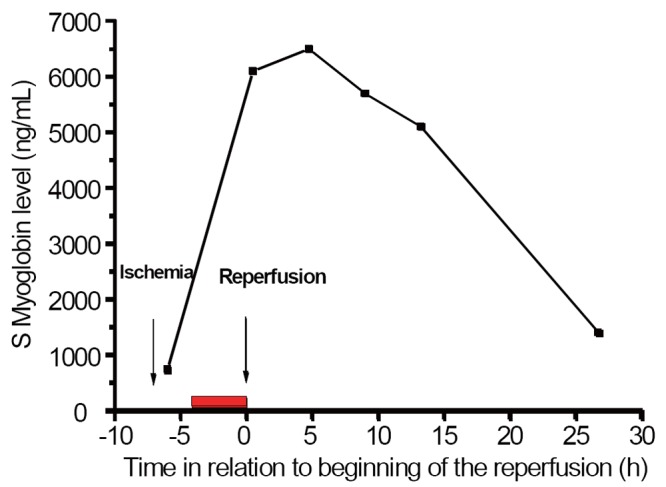
The 24 hours serum myoglobin levels in our patient. Time with zero representing start of reperfusion. The red bar is the time interval of preoperative cooling.

Postoperative respirator time was 5.5 hours. 2250 mL fluid were infused over 12 hours achieving a urinary output of 190 mL/h. The acidosis was normalized twelve hours postoperatively. S-Creatinine one month before admission was 87 mmol/L. Just before surgery this value was 547 mMol/L but had returned to normal 72 hours postoperatively. The first S-Myoglobin 30 minutes postoperatively was 6100 ng/L and was checked every four hours for the first thirteen hours showing a decline to 1400 ng/L 27 hours after reperfusion (Figure 2).

Postoperatively, the superficial skin temperature of the right foot was 30°C for the first five hours and then gradually increased to 35.5°C. The temperature of the left foot decreased from 29.7°C to 27°C over the first five hours then increased to 35°C eleven hours later.

## DISCUSSION

The main observation in this case was that the application of topical hypothermia while waiting for surgery was able to protect severely ischemic skeletal muscle from developing permanent damage. Topical hypothermia was first described to protect the myocardium during periods of ischemia and has been used since the early days of cardiac surgery (1-3). The clinical findings in our case are consistent with the experimental results regarding the protective role of pre-ischemic hypothermia in plastic surgery where musculo-cutaneous flaps (4, 5) or isolated gracilis muscle (6) have been studied. Wright used post-ischemic hypothermia to diminish ischemic damage in an experimental model (7). Petrasek used a small animal model to study limb ischemia (8). Despite promising experimental data regarding the protective effect of hypothermia on skeletal muscle, topical hypothermia has not been described in clinical practice.

A drawback in our case is that we cannot really say anything about the effectiveness of the cooling process as we did not measured the temperature of the muscles in the hypothermic lower leg.

The successful outcome in this case was, of course, also due to gradual reperfusion (9) caused by the restoration of profunda flow, followed by restoration of flow in the SFA, poplitea and lower leg arteries some 30-60 minutes later.

However, in a consecutive cohort of 33 patients with acute ischemia TASC type IIB/III at our department over the last 5 years, the 30-day mortality was 27% and amputation frequency 6%. Of these, 17 patients had a median maximum myoglobin level after restoration of blood flow of 12 000 ng/ml (94000-1500). An extreme low value was excluded (revascularization within 2 hours and myoglobin level 670 ng/ml). If cases with somewhat milder ischemia and longer duration (12-36 h) are excluded, patients had a mean myoglobin level >11 000 ng/ml. The patient that we describe would certainly qualify for the severe group. With a maximum post-revascularization myoglobin level of less than 8 000 ng/ml in our case, compared to levels of 11-94 000 in comparable cases without hypothermia at our department, we feel that the application of cooling bags led to a more favorable outcome than in the “normal” TASCIIb/III case.

This case concerned a patient on the ward with intensive care and monitoring. The positive outcome leads us to speculate on the future role of topical hypothermia in the field of prehospital care. It is not unusual for trauma victims to suffer ischaemia of an extremity secondary to vessel injury or fracture, in such cases topical hypothermia could win many valuable minutes of viability while awaiting transport and surgery. The same applies to combat situations or mountain rescue scenarios where prolonged ischaemia is anticipated due to delayed evacuation.

In conclusion preoperative topical hypothermia protected the limb muscles against serious ischemia in terms of serum myoglobin dynamics and muscular function, and may be a promising way to treat patients with TASC class IIb/III ischemia pending revascularization. This remains to be seen in a prospective randomized study.
